# Transcriptomic profiling in muscle and adipose tissue identifies genes related to growth and lipid deposition

**DOI:** 10.1371/journal.pone.0184120

**Published:** 2017-09-06

**Authors:** Xuan Tao, Yan Liang, Xuemei Yang, Jianhui Pang, Zhijun Zhong, Xiaohui Chen, Yuekui Yang, Kai Zeng, Runming Kang, Yunfeng Lei, Sancheng Ying, Jianjun Gong, Yiren Gu, Xuebin Lv

**Affiliations:** 1 Animal Breeding and Genetics Key Laboratory of Sichuan Province, Sichuan Animal Science Academy, Chengdu, Sichuan, China; 2 Chengdu Biotechservice Institute, Chengdu, Sichuan, China; Universitat de Lleida, SPAIN

## Abstract

Growth performance and meat quality are important traits for the pig industry and consumers. Adipose tissue is the main site at which fat storage and fatty acid synthesis occur. Therefore, we combined high-throughput transcriptomic sequencing in adipose and muscle tissues with the quantification of corresponding phenotypic features using seven Chinese indigenous pig breeds and one Western commercial breed (Yorkshire). We obtained data on 101 phenotypic traits, from which principal component analysis distinguished two groups: one associated with the Chinese breeds and one with Yorkshire. The numbers of differentially expressed genes between all Chinese breeds and Yorkshire were shown to be 673 and 1056 in adipose and muscle tissues, respectively. Functional enrichment analysis revealed that these genes are associated with biological functions and canonical pathways related to oxidoreductase activity, immune response, and metabolic process. Weighted gene coexpression network analysis found more coexpression modules significantly correlated with the measured phenotypic traits in adipose than in muscle, indicating that adipose regulates meat and carcass quality. Using the combination of differential expression, QTL information, gene significance, and module hub genes, we identified a large number of candidate genes potentially related to economically important traits in pig, which should help us improve meat production and quality.

## Introduction

Pig meat is an important source of protein for humans. Meat quality is mainly determined by higher intramuscular fat (IMF) and monounsaturated fatty acid (MUFA) contents [1], which influence the water-holding capacity, fat content, juiciness, and flavor of the meat and are relevant to the obesity of humans [2]. Adipose, liver, and skeletal muscles are the most important tissues and organs associated with whole-body lipid metabolism [3,4], which determine the IMF content and fatty acid (FA) profile in muscle. Adipose is a metabolic and endocrine tissue that acts in *de novo* FA synthesis and as a fat storage depot, which is involved in circulating free FA and regulating lipid metabolism [5]. It produces and releases adipokines, which are involved in the maintenance of metabolic homeostasis and play an important role in the development of diseases associated with obesity [6].

The IMF and MUFA levels of pigs are determined by both genetics and the diet [2,7]. Chinese indigenous breeds (referred to hereafter as Chinese breeds) display a lower growth rate, higher fat percentage, and lower muscle mass, but better meat quality, than Western pig breeds such as Yorkshire (YY). Having been under intensive selection for improving growth rate and muscularity, this latter breed typically shows rapid growth and a high lean meat percentage [8]. Clarification of the genetic differences between these two pig groups should provide insight into the genetic architecture of complex traits such as fat deposition, metabolism, and growth.

By comparing the transcriptome of skeletal muscle and adipose tissue from Chinese breeds and Western pig breeds, the underlying molecular mechanisms have started to be uncovered. For example, several studies have been carried out on *longissimus dorsi* (LD) muscle [9–14] and subcutaneous adipose tissue [15]. Several studies have also been performed on breeds with extreme phenotypic differences, or on other breeds with similar phenotypes in subcutaneous adipose tissue [4,16–19] and LD muscle [20–24]. However, two issues remain to be investigated: 1) Although muscle and adipose tissues are all important for meat quality, they have mainly been investigated separately, and no studies have compared these two tissues simultaneously. 2) Previous studies only focused on one to three breeds among Lantang, Tongcheng, Tibetan, Wuzhishan, and Rongchang [9,10,13,14,25], but many other Chinese breeds have not been investigated, such as Chenghua (CH) and Neijiang (NJ). The differences in phenotypic traits and levels of gene expression between these Chinese breeds and Western commercial breeds remain unclear.

Weighted gene coexpression network analysis (WGCNA) has been widely used to couple coexpression modules to phenotypic measurements [26,27]. This method has been used to study muscle development [28,29] and obesity [18,30], but rarely to identify coexpressed genes associated with meat quality [31], and the difference of how strongly adipose and muscle tissues are linked to meat quality is still unknown.

To explore the molecular mechanism behind the phenotypic differences between Chinese breeds and Western pigs, in this study, we selected seven Chinese breeds, namely, CH, NJ, Tibetan pig (TP), Qingyu (QY), Wujin (WJ), Yacha (YC), and Yanan (YN), and one introduced Western breed, YY. In these breeds, we measured important phenotypic traits including growth performance, carcass quality, meat quality, and amino acid composition. We also investigated the transcriptome of adipose and LD muscle tissues for individuals for which data on the phenotypic traits were available. Combining these two data sets, with the aid of differential gene expression analysis and WGCNA, we have the opportunity to systematically assess the potentially common molecular regulatory mechanisms that underlie the huge phenotypic differences between these two groups of breeds. Furthermore, interpretation of the complex phenotypic differences among pigs also promotes our understanding of associated traits in humans, such as growth, obesity, and metabolism [32].

## Results

### Phenotypic variation among pig breeds

In this study, 3 growth performance traits, 58 carcass quality traits, 7 meat quality traits, 18 amino acid composition traits, and 15 fat acid composition traits were measured in the two groups of breeds, namely, seven Chinese breeds and the YY breed (S1 and S2 Tables). As shown in S1 Fig) similar traits were clustered together, such as the traits of skin, fat, amino acid composition, lean meat and bone, and carcass weight; and 2) YY has significantly higher contents of lean meat and bone, and carcass weight, while the Chinese breeds have higher skin and fat contents. In addition, YY has a significantly lower feed gain ratio (FGR) and higher average daily gain (ADG), indicating that it is suitable for meat production. We found that YY also has lower meat quality traits, such as PH1 (45 min postmortem), IMF, and marbling (MBL), which explains why YY’s meat is not particularly sought after by consumers. Regarding the amino acid composition, YY has higher content of threonine and lower contents of methionine and proline. Regarding the FA composition, YY has higher polyunsaturated fatty acid (PUFA) and lower MUFA, but does not differ from the Chinese breeds in terms of saturated fatty acid (SFA). We also identified major variations of traits among the Chinese breeds, such as in growth performance traits such as FGR and body length (BL); in carcass quality traits such as slaughter weight (SW) and loin muscle area (LMA); and in meat quality traits such as meat color (CLR), MBL, and levels of amino acids such as aspartic acid and tyrosine. These findings are consistent with the phylogenetic analysis showing that the Chinese breeds’ group is more heterozygous than its counterpart in Europe [33]. These phenotypic differences in fat and skin content, lean meat content, meat quality, and growth performance between breeds imply intrinsic differences in gene expression.

**Fig 1:**
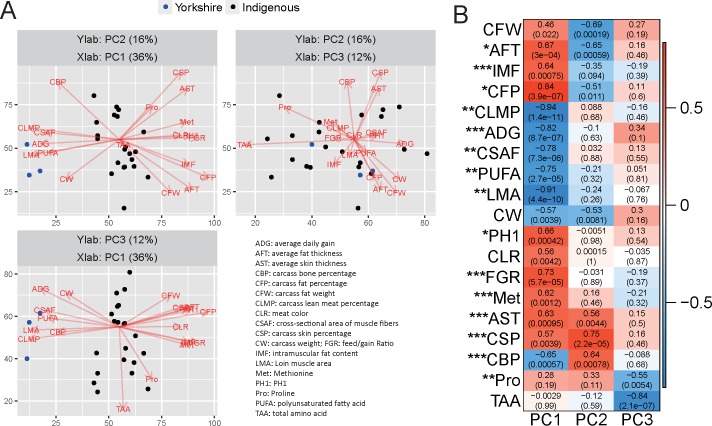
PCA analysis on the 101 phenotypic traits of the pigs. (A) Bi-plot of the loadings and scores of the three PCs. For clarity, traits in red vectors are those with a significance level of the correlations between traits and the loadings < 0.01, and further selected for the representative traits. Trait abbreviations are provided in the lower right panel. Blue and black dots represent YY and Chinese breeds, respectively. (B) Correlations of the presented phenotypes to the loadings of the three PCs. Pearson’s correlation coefficients (the numbers outside parentheses) and significant *P* values of the correlation (within parentheses) are provided in the cell; red represents a positive correlation and blue represents a negative one. Asterisks before the traits indicate the significance of the difference between the Chinese breeds and YY. * *P*<0.05; ** *P*<0.01; *** *P*<0.001.

Principal component analysis (PCA) on the 101 correlated phenotypic traits extracted three phenotypic gradients, PC1, PC2, and PC3, representing 36%, 16%, and 12% of the explained variance, respectively. YY pigs can be distinguished from Chinese breeds by PC1 (Fig 1). We found that the IMF, weight of fat, skin thickness, fat thickness, and methionine level were positively correlated with PC1, while PUFA, and the weights of lean meat and bone were negatively correlated with PC1. Although the weights of fat and fat thickness were also positively correlated with PC2, their significance levels were lower than for PC1. Interestingly, the skin thickness was negatively correlated with PC2. No phenotypes were found to be positively correlated with PC3, but significant negative correlations between the amino acid content and PC3 were identified. A full list of the phenotypic traits with Pearson’s correlation *r*>0.3 and significance *P*<0.01 is provided as S2 Fig. Taking these findings together, PC1, the main PC to explain the phenotypic differences between these pig breeds, can split the traits into two groups: one is positively correlated with PC1 and superior in Chinese breeds, and the other is negatively correlated with PC1 and superior in the YY breed.

### Sequencing and mapping

After filtering the adaptor and low-quality reads, 244.7 Gb of clean data was obtained for 48 samples, namely, two tissues from eight pig breeds with three replicates of each sample. Three samples, NJ_66_Muscle, CH172_Muscle, and YN163_Muscle, were removed from further analysis because of an unexpected clustering position or low correlation with the replicates. Therefore, there are two replicates for muscles from the NJ, CH, and YN breeds. The clean data for each sample ranged from 4.7 to 5.2 Gb. The clean reads accounted for 84.9% to 96.7% of the raw reads. The Wuzhishan pig (WZSP) genome reference [34] was used to align the RNA-seq reads and the total mapped reads accounted for 73.4% to 88.1% of the total clean reads, and the unique mapped reads ranged from 54.2% to 76.2%. We also calculated the same measurements using Sscrofa10.2 as a reference; the total mapped reads and the unique mapped reads were slightly inferior to those of WZSP. Therefore, we used WZSP as a reference for the subsequent analysis (S3 Table). Reads per kilobase per million reads (RPKM) was used as a measurement of the expression level. Of the 20,326 WZSP official genes, 55.1% to 58% have expression level RPKM>1 across most muscle tissues, while 58.1% to 61.0% have RPKM>1 across all adipose tissues, which indicates that the samples of the same tissue from various breeds covered similar numbers of genes (S4 Table). In addition, the total numbers of genes with expression level RPKM>1 in muscle and adipose tissues were 13,973 (68.8%) and 14,287 (70.3%), respectively, which indicates that each breed has numerous genes specific to it alone, which are potentially responsible for the specific phenotypes. Furthermore, the genes commonly expressed among the different breeds in the muscle and adipose tissues numbered 10,312 (50.7%) and 9,582 (47.1%), respectively. Clustering analysis was performed using all of the expressed genes with the log2-transformed RPKM after adding 1. All of the samples were grouped into two clusters representing muscle and adipose tissues (S3 Fig), although the replicates were not completely clustered together, which may have been caused by the large variation in the sampling procedure. All of the Pearson’s correlations between the replicates were greater than 0.95, which indicates the high quality of the sequencing (S4 Fig).

### Differential gene expression analysis and qPCR validation

**Fig 2:**
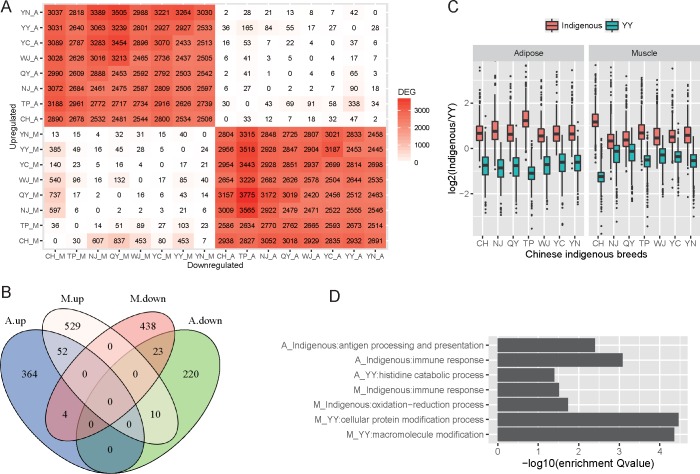
Consistent expression levels between Chinese indigenous breeds and the YY breed. (A) The numbers of DEGs between all pairs of samples. The numbers indicate the genes that were upregulated for the samples on the y-axis and downregulated on the x-axis. The darker the red, the greater the number of DEGs. A: adipose tissue; M: *longissimus dorsi* muscle tissue. (B) Venn diagram summarizing the number of DEGs shared among tissues. (C) Comparisons of the expression level between the DEGs that were highly expressed in YY and in Chinese breeds for the two tissues. The ratio is calculated based on the average RPKM of the replicates. Red indicates the genes expressed more highly in Chinese breeds and blue indicates those expressed more highly in YY. (D) The selected enriched biological processes in the Gene Ontology gene sets for the upregulated genes in the two tissues from Chinese breeds and the YY breed.

In this study, the genes that were differentially expressed between the Chinese breeds and YY were identified. In adipose tissue, 673 differentially expressed genes (DEGs) were found, with 420 being highly expressed in Chinese breeds and 253 in YY, without any DEGs being shared between the upregulated and downregulated genes across the Chinese breeds (Fig 2A and 2B). The largest number of DEGs was found from the comparison between YY and TP, with 165 presenting higher expression in YY and 338 in TP. The values of weight-related variables were lower in TP as it is the lightest of the breeds. Interestingly, the level of stearic acid and the cross-sectional area of muscle fibers were higher in YY than in TP (Student’s *t*-test, *P* = 1.2e^−4^ and 1.8e^−3^, respectively), and proline, carcass fat percentage (CFP), and hind leg fat percentage were higher in TP than in YY (Student’s *t*-test, *P* = 9.4e^−4^, 6.7e^−3^, and 6.7e^−3^, respectively); the findings for this breed were the most significant upon comparing all seven Chinese breeds with YY (S5 Table), which may be explained by the fact that the number of DEGs between YY and TP is the highest. In muscle tissue, 1,056 DEGs were found, with 591 being highly expressed in Chinese breeds and 465 in YY. Furthermore, the different Chinese breeds also do not share common DEGs between the upregulated and downregulated genes. What is different in the case of muscle is that the largest number of DEGs was identified for the comparison between CH and QY. By comparing the phenotypic features between CH and all other breeds, we found that the level of tetradecanoic acid was higher in CH than in QY (Student’s *t*-test, *P* = 4.5e^−3^) and cysteine content was higher in QY than in CH (Student’s *t*-test, *P* = 0.01), which were also the variables with the most significant differences among the comparisons between CH and the other pig breeds (S6 Table). This may have contributed to the largest number of DEGs having been identified in this case.

More than 86% of DEGs were tissue-specific, and only 52 upregulated and 23 downregulated DEGs were shared between the two tissues in Chinese breeds (Fig 2B). We also detected 43 genes related to lipid biosynthesis, transport, and metabolism (S7 Table).

To validate the accuracy of the expression level measured by RNA sequencing (RNA-seq), we randomly selected five DEGs (GJA9, FAAH, SLC2A5, CCL24, and IYD) for qPCR (S8 Table). These five DEGs include genes upregulated and downregulated across all or several breeds in the two tissues. The overall correlation between the RPKM and the qPCR is 0.5 (*P* = 2.5e^−15^), and 72% of DEGs showed a consistent direction of differential expression (S5 Fig), which indicates that the DEGs identified using RNA-seq were reliable.

### Functional analysis of DEGs

Because the Chinese breeds share similar levels of meat quality, we postulated that the DEGs in one breed would exhibit similar expression trends in the other breeds within this group compared with YY. As expected, the genes upregulated in Chinese breeds were expressed more highly across all Chinese breeds than in YY, and vice versa, for both adipose and muscle tissues (Fig 2C). The consistent expression level of the Chinese breeds compared with YY indicates that all of the DEGs detected across the Chinese breeds contribute to their distinct meat quality, albeit potentially having various levels of effect in the different breeds.

In the next phase of this study, we investigated the functions of all of the DEGs detected from all breeds together. Gene Ontology (GO) analysis of the DEGs indicated that the highly expressed genes in YY adipose tissue were mainly involved in histidine catabolic process, while the highly expressed genes in Chinese breeds were associated with GO terms including oxidoreductase activity, immune response (e.g., antigen processing and presentation, and MHC protein complex), metabolic process (e.g., small-organism metabolic process, acyl-CoA metabolic process, and cofactor metabolic process), biosynthetic process (e.g., acetyl-CoA biosynthetic process), and muscle development (e.g., skeletal muscle tissue development and muscle structure development). In muscle tissue, the enriched GO terms of highly expressed genes in YY included transport process (e.g., phosphotransferase activity, cytoskeletal protein binding, and transferase activity), protein modification (e.g., protein phosphorylation, macromolecule modification, and protein kinase activity), and cytoskeleton organization. Highly expressed genes in Chinese breeds were mainly involved in oxidation reduction (e.g., oxidoreductase activity and oxidation–reduction process), immune response, mitochondria (e.g., organelle envelope and organelle inner membrane), catalytic activity, and cofactor binding (Fig 2D and S9 Table).

KEGG pathway analysis of the biased genes in these breeds also provided some interesting findings (S10 Table), most of which were consistent with the results from the GO analysis. In adipose tissue, focal adhesion, cytokine–cytokine receptor interaction, and melanogenesis were identified as the main categories associated with the genes upregulated in YY, while lysosome, pyruvate metabolism, antigen processing and presentation, and osteoclast differentiation were closely related to the genes upregulated in Chinese breeds. In muscle tissue, the genes upregulated in YY were mainly associated with transcriptional misregulation in cancer, signaling pathways regulating pluripotency of stem cells, and the insulin signaling pathway. The genes upregulated in Chinese breeds were mainly those associated with herpes simplex infection, drug metabolism, Parkinson’s disease, glycosylphosphatidylinositol-anchor biosynthesis, and apoptosis.

We further narrowed down the DEGs according to how many breeds in which they were identified upon comparisons of the Chinese breeds with YY. Interestingly, we found that 181 and 161 genes were differentially expressed across at least two breeds in adipose and muscle tissues, respectively (S11 Table). Among these, 27 and 10 genes were differentially expressed across at least five breeds in the two tissues, respectively (S11 Table).

### Comparison of DEGs with a QTL database

To define the functions of the DEGs, we mapped them to the pigQTLdb. Interestingly, we found that DEGs were significantly enriched for QTL regions (Fisher’s exact test, *P* = 0.04), and 163 DEGs overlapped with QTL regions linked to meat, carcass, and production traits; in total, 183 unique QTL traits were localized to factors encoded at 135 unique regions. Among these QTL-related genes, 29 and 42 were downregulated and upregulated in adipose tissue of Chinese breeds, while in LD muscle tissue, the numbers were 45 and 57, respectively (S12 Table). Among the 163 DEGs, 66 genes clustered in 28 QTL regions; the largest number of genes in a cluster was 5. The cluster with the most genes was Cluster 13, located on chr6:57886136–59136767, which harbored *PLCH2*, *CALML6*, *C1ORF70*, *ATAD3*, and *UBE2J2*. Among these unique QTL regions, one region, Chr2:6947966–7124118, was shown to be associated with seven unique traits, namely, arachidonic acid content, cis-vaccenic acid content, fatty acid C20:3 to C18:2 ratio, intramuscular fat content, myristic acid content, palmitoleic acid content, and stearic acid content. Two genes from Cluster 16, *FERMT3* and *STIP1*, related to this region were differentially expressed. The clustering of these trait-related genes in a single region may have benefits in terms of trait regulation. We also found that, among the 183 unique QTL traits, drip loss and loin muscle area were related to the greatest number of genes (*n* = 16). The drip loss trait is related to the following genes: *ST8SIA2*, *ALG2*, *FAM166B*, *KANK1*, *CD24*, *ST6GALNAC4*, *TBX18*, *TPM1*, *MNS1*, *WZSP017628*, *WDR32*, *TLE4*, *C9ORF4*, *C9ORF5*, *CTNNAL1*, and *EXD1*. Among these 16 genes, 15 are located on chromosome 1, with 5 being clustered into Clusters 8 and 27. The 16 genes identified for the trait of loin muscle area were *SLC30A4*, *ALDH4A1*, *STX3*, *MRPL16*, *APITD1*, *NPC1*, *MIER3*, *TTC9*, *PCNX*, *HSPA5*, *KLHL31*, *HES1-A*, *NAAA*, *UNQ1879/PRO4322*, *WZSP017749*, and *WZSP018259*. Eight of these were clustered into three clusters, namely, Clusters 11, 17, and 25 (S12 Table).

### Coexpression network construction of the two tissues

A weighted coexpression network was constructed using the WGCNA method (S6 Fig). For the adipose and muscle tissues, 23 and 20 coexpression modules were detected, respectively (S7 Fig). The quality of the two coexpression networks was assessed by the *modulePreservation* function in WGCNA, which revealed that the quality of the modules from the two networks was high, with quality values >10; and the quality of two modules was at an intermediate level, with values between 2 and 10, for one adipose module and one muscle module (S8 Fig). Comparing the two networks, the level of module preservation was high or intermediate, with a Z_*summary*_ value >2, except for three modules in adipose and four modules in muscle tissue (S9 Fig). These results are also consistent with the chi-square test of the significance of the overlap between the modules from adipose and muscle, where almost every module in muscle can be covered significantly by at least one module in adipose (S10 Fig).

### Biological significance of network modules

**Fig 3:**
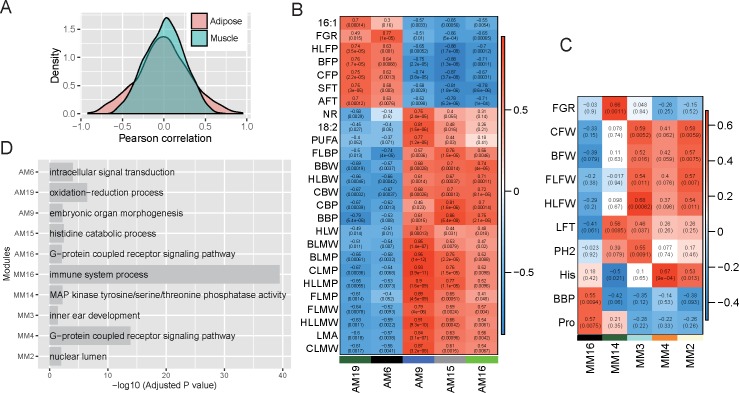
Higher correlation between adipose modules and traits than for muscle modules. (A) The distribution of Pearson’s correlation between the module eigengene and traits for adipose and muscle tissues. (B) The module eigengene significance of adipose tissue. The displayed traits and modules have at least Pearson’s correlation *r*>0.7 and *P*<0.01. Cell color represents the correlation (red, positive correlation; blue, negative correlation, according to the color legend). Two numbers in each cell show the correlation level (upper) and correlation significance (lower in the parentheses). Rows represent traits and columns represent coexpression modules. (C) The module eigengene significance of muscle tissue. The displayed traits and modules have at least Pearson’s correlation *r*>0.5 and *P*<0.01. The cell color, numbers in each cell, and X and Y axes have the same meanings as those in B. (D) The enriched Gene Ontology biological processes for the selected modules in adipose and muscle tissues. The height of the bar corresponding to the minus log10-transformed adjusted *P* value. A: adipose, M: muscle.

By comparison with the phenotypic traits, we could detect which modules influenced the phenotypic variation. Surprisingly, we found that adipose tissue has a markedly stronger association with the phenotypic traits than muscle. The numbers of comparisons for which Pearson’s correlation (r) was >0.5 were 140 and 26 in adipose and muscle, respectively (chi-square test, *P* = 9.1e^−15^, Fig 3A, S11 and S13 Figs). Upon applying cut-offs of Pearson’s correlation *r*>0.5 and *P*<0.01, 16 modules and 53 traits (including PC1) were identified to be significantly correlated in adipose (S12 Fig), while 5 modules and 10 traits were in muscle (Fig 3C). Among the 10 traits in muscle, 6 matched those in adipose, namely, FGR, back waist bone percentage (BBP), lumbosacral fat thickness (LFT), back waist fat weight (BFW), carcass fat weight (CFW), and hind leg fat weight (HLFW). A major contrast was identified in that adipose has 47 unique correlated traits, while muscle only has 4.

All of the adipose modules that significantly correlated with traits could be clearly classified into two groups. The first module group was positively correlated with the fat deposit phenotypic traits, such as the carcass fat percentage (CFP), average fat thickness (AFT), intramuscular fat content (IMF), PC1, and FGR, including the modules AM19, AM5, AM4, AM6, and AM8. AM2 and AM1 only highly correlated with leucine composition and drip loss, respectively, and weakly correlated with the other traits. The second module group was positively correlated with lean meat percentage and PUFA, including the modules AM9, AM15, AM16, AM23, AM10, AM13, AM14, AM11, and AM12. The positively correlated traits of these two module groups showed hardly any overlap (S12 Fig and Fig 3B). For simplicity, a correlation heatmap of the 5 modules and 26 traits with Pearson’s correlations *r*>0.7 was produced for adipose (Fig 3B).

The first module group was mainly associated with oxidation–reduction process (AM19), intracellular signal transduction (AM6), response to external stimulus (AM5), tetrapyrrole biosynthetic process (AM4), and defense response to virus (AM8). The second module group mainly represented embryonic organ morphogenesis (AM9), histidine catabolic process (AM15), G-protein-coupled receptor signaling pathway (AM16), cellular macromolecule metabolic process (AM23), cell surface receptor signaling pathway (AM13, AM14), and protein retention in ER lumen (AM11) (Fig 3D and S13 Table).

In muscle, three modules positively correlated with fat weight (MM3, MM2, and MM4), one module positively correlated with BBP and proline content (MM16), and the other one positively correlated with FGR and lumbosacral fat thickness (LFT) (MM14). The GO analysis revealed that muscle modules that significant correlate with the phenotypic traits are mainly involved in immune system process (MM16), inner ear development (MM3), nuclear lumen (MM2), G-protein-coupled receptor signaling pathway (MM4), and MAP kinase tyrosine/serine/threonine phosphatase activity (MM14) (Fig 3D and S13 Table).

### Modules correlated with phenotypes also enriched for DEGs

We found that the modules significantly correlated with the phenotypic traits were also enriched for the DEGs between Chinese breeds and YY, and the modules that positively correlated with the traits with higher values in YY were only enriched for the DEGs upregulated in YY itself, the same for the modules that positively correlated with the traits with higher value in Chinese breeds (hypergeometric test, Table 1). For example, AM4, AM5, AM6, AM19, MM3, and MM14 only overlapped with the genes upregulated in Chinese breeds, while AM9, AM10, AM11, AM12, AM13, AM14, AM15, and AM16 only overlapped with the genes upregulated in YY. These results indicate that the modules correlated with the phenotypic variation were potentially caused by the DEGs.

**Table 1:** Modules significantly correlated with phenotypic traits that were also significantly enriched for DEGs.

Module	Module size	#DEG	P1	#Up	P2	#Down	P3
AM19	440	66	0	66	0	0	1
AM5	75	11	0.00016	11	1.40e^-6^	0	0.73
AM4	1700	120	7.70e^-8^	120	0	0	1
AM6	530	120	0	120	0	0	1
AM2	65	3	0.35	3	0.12	0	0.68
AM1	21	0	0.63	0	0.46	0	0.31
AM8	32	1	0.44	1	0.24	0	0.43
AM13	210	18	0.0046	4	0.74	14	4.80e^-6^
AM14	84	6	0.094	1	0.7	5	0.0035
AM11	160	7	0.48	0	0.99	7	0.0076
AM12	96	5	0.29	0	0.94	5	0.0067
AM23	3500	94	1	14	1	80	0.0031
AM15	100	25	5.70e^-13^	0	0.95	25	0
AM16	380	26	0.017	0	1	26	5.40e^-10^
AM9	95	40	0	0	0.94	40	0
AM10	100	13	0.00022	0	0.95	13	2.30e^-9^
MM14	97	9	0.21	9	0.0087	0	0.96
MM3	30	4	0.076	4	0.0084	0	0.64
MM4	770	26	1	22	0.98	4	1
MM2	110	6	0.75	6	0.19	0	0.98
MM16	670	28	1	21	0.92	7	1

Note: The significance of the overlapped DEGs was tested using the hypergeometric test for the total DEGs (P1), genes upregulated in Chinese breeds (P2), and genes upregulated in YY (P3). Indig: Chinese breeds.

### Hub gene analysis of the significant phenotypic trait-related modules

To find the key drivers of the phenotypic variation, we took advantage of the phenotype-related modules and performed an analysis of the hub genes. For each module, the top 10% of nodes in terms of membership of the most modules were considered as hub genes. In total, 761 and 168 genes were found in the adipose and muscle modules with significant trait correlation, respectively. Among the hub genes, 70 and 13 genes were associated with QTL regions and 102 and 7 genes were DEGs, in adipose and muscle tissues, respectively (S15 Table). Functional analysis of the hub genes indicated that the translational activity was enriched for the adipose hub genes, while muscle hub genes were mainly associated with protein binding (S16 Table).

**Fig 4:**
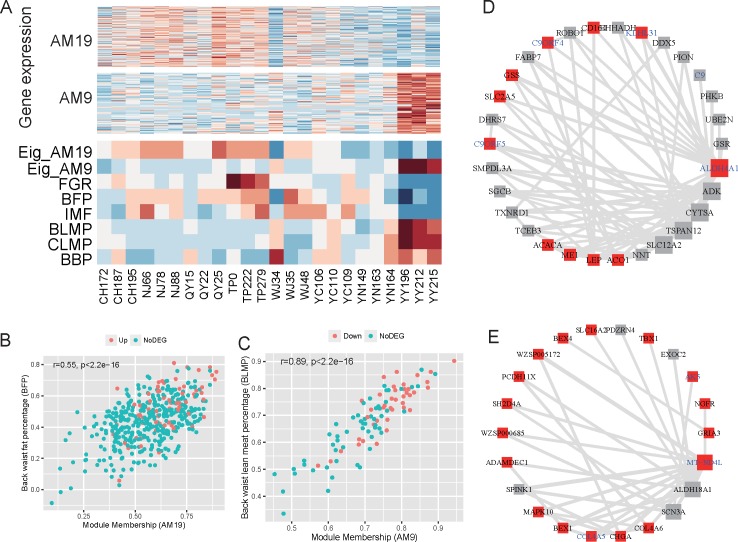
Adipose modules AM19 and AM9 are significantly correlated with the fat and lean meat phenotypic traits. (A) The expression and eigengene values of modules AM19 and AM9 have opposite correlations with the phenotypic traits. Each row in the heatmaps was normalized, with red denoting a higher Z score of the expression (measured using RPKM) or the phenotypic measurements, and blue denoting a lower one. In the expression section, each row represents one gene. Eig_AM19 and Eig_AM9 represent the module eigengenes of AM19 and AM9, respectively. Each column represents one individual pig. FGR: feed/gain ratio, BFP: back waist fat percentage, IMF: intramuscular fat content, BLMP: back waist lean meat percentage, CLMP: carcass lean meat percentage, BBP: back waist bone percentage. (B) Gene significance of back waist fat percentage is positively correlated with the module membership in AM19. Red denotes upregulation in Chinese breeds. (C) Gene significance of back waist lean meat percentage is positively correlated with module membership in AM9. (D) The network of the top 30 genes with the highest module membership in module AM19; only the edges with topological overlap above a threshold of 0.05 are displayed. The rectangle represents the DEGs between Chinese breeds and Yorkshire. (E) The network in module AM9 with the same criteria as those in D. The coexpression networks reflect the degree of topological overlap (the size of the vertex), the DEGs (the red color of the vertex), and the QTL association (the blue color of the label).

In Fig 3, the adipose modules are clearly split into two classes with opposite correlations to the phenotypic traits. To illustrate the relationship between the genes in the modules and the traits, and the DEGs and QTL information together, for further analysis, we selected the modules AM19 and AM9, which displayed the most significant correlations with the traits, as representatives to identify the key drivers of the trait differences. We first compared the expression value to the phenotypic traits across the 24 analyzed pig individuals. As expected, the expression of AM19 was positively correlated to FGR and the fat traits, such as BFP and IMF, while AM9 positively correlated to the bone and lean meat traits, such as BLMP, CLMP, and BBP (Fig 4A). The variable gene significance (GS) measured the level of the relationship between the gene expression and the traits. In addition, the variable module membership (MM) was used to represent the correlation of the gene to the module eigengene. The higher the module membership, the greater the likelihood of the gene being the hub gene of the module, thereby playing a key role in the function of the module. Significant correlations between the GS and MM were observed for the traits BFP and MM in module AM19 (Pearson’s correlation, *r* = 0.55, *P*<2.2e^−16^; Fig 4B) and the trait BLMP in module AM9 (Pearson’s correlation, *r* = 0.89, *P*<2.2e^−16^; Fig 4C). Hub gene analysis based on the MM in AM19 detected *ALDH4A1*, *ADK*, *CYTSA*, *TSPAN12*, and *SLC12A2* as hubs (Fig 4D). In AM9, *MT-ND4L*, *ALDH18A1*, and *SCN3A* were detected as hubs (Fig 4E). Among these genes, *ALDH4A1* and *MT-ND4L* are not only differentially expressed between Chinese breeds and Yorkshire, but also associated with meat and carcass QTL regions.

In muscle tissue, MM16 and MM3 were selected as representative modules to detect the driver hub genes related to proline level and fat weight (S14A–S14C Fig). The network analysis detected *LAMP3*, *IGHG2*, *WZSP018232*, and *PLA2G2D* as hub genes in module MM16 (S14D Fig). In MM3, hub genes *WZSP006812*, *SCGB1D2*, *SCGB2A2*, *PLUNG*, *WZSP017782*, and *WZSP017789* were fully connected with each other and the intensity of the connection among these hub genes was greater than that to other genes, which indicates that these genes undertake close cooperation (S14E Fig).

### Trait-related genes combining the GS and QTL data

Although we detected the modules that were positively correlated with phenotypic traits, most of the modules were simultaneously correlated with more than one trait. To identify trait-specific genes, we also determined the top 10 genes according to the variable of GS for each phenotypic trait in adipose and muscle, and set 0.5 as the cut-off for this variable (S18 Table). These genes may contribute to the phenotypic differences between Chinese and YY breeds. Although the module trait correlation showed a marked difference between adipose and muscle, these top 10 genes displayed similar GS values between the two tissues. Among these genes, 26 and 23 genes appeared for more than five traits in adipose and muscle, respectively. The gene showing associations with the largest numbers of traits in both adipose and muscle was *MT-ND4L*, at 27 and 25, respectively (S18 Table).

We also detected genes with high GS with the phenotypic trait (*r*>0.4) (referred to hereafter as GS evidence) and that were simultaneously associated with the trait-related QTL region (referred to hereafter as QTL evidence). We found 65 genes for 19 traits in adipose and 42 genes for 16 traits in muscle that satisfy these criteria (S17 Table). Among these traits, ADG, IMF, and AFT had the most correlated genes, namely, 19, 7, and 7, respectively, in adipose. In muscle, drip loss (DL) and ADG had the most correlated genes, namely, 16 and 6, respectively.

## Discussion

In our study, we investigated 101 phenotypic traits in seven Chinese indigenous pig breeds and one Western commercial breed (YY). Comparative statistical analysis revealed that Chinese breeds have distinct traits regarding growth performance, carcass quality, and meat quality, and they were clearly differentiated from YY by PC1 in the PCA. The values of the phenotypic traits that positively correlated with PC1 are superior in the Chinese breeds, and those negatively correlated with PC1 are superior in the YY breed. This implies that, although there is large phenotypic variation among the Chinese breeds, they have consistent phenotypic differences when compared with the Western commercial breed, a finding that provided a foundation for the subsequent DEG analysis. As expected, the DEGs expressed highly in one Chinese breed were also expressed highly across all other Chinese breeds, which was the same for the genes expressed at a low level, indicating that there are similar underlying regulatory pathways between the different Chinese breeds and YY. Association analysis between the coexpressed modules and the phenotypic traits revealed a similar pattern whereby the modules are split into two groups, one positively correlated with the traits representing the Chinese breeds and the other with the traits representing the YY breed. To discover the trait-specific correlated genes, we also detected the genes with the highest level of gene significance for the traits and the genes related to the traits given the evidence for both QTL and gene significance. To the best of our knowledge, this is the first report of a study simultaneously investigating the relationship between phenotypic traits and gene expression in both adipose and muscle [18,31,35]. Based on the above-mentioned analysis, we will discuss how factors expressed in adipose and muscle tissues regulate growth performance, carcass quality, and meat quality.

Two growth performance traits displayed significant differences between the Chinese breeds and YY, namely, average daily gain (ADG) and feed/gain ratio (FGR), with ADG being higher in the YY breed and FGR being higher in the Chinese ones. Because no specific modules are correlated with ADG or FGR, we focused on the genes in the top 10 in terms of the level of GS and the genes with both QTL and GS evidence. Of the 19 genes with ADG QTL and GS evidence in adipose, six are involved in metabolic process, namely, *PLA1A*, *INPP5B*, *DENR*, *PLCB4*, *ICK*, and *RAD23B*, including lipid metabolic (*PLA1A*, *INPP5B*, and *PLCB4*) and cellular macromolecule metabolic (*DENR*, *ICK*, and *RAD23B*). Four participate in signal transduction: *INPP5B*, *GPR81*, *PLCB4*, and *PREX2*. Another four have nucleic acid binding function: *ZCCHC8*, *DENR*, *ZCCHC2*, and *RAD23B*. Among these genes, *ZCCHC2* and *PREX2* are downregulated in adipose and muscle, respectively. In muscle, six genes were identified with both ADG QTL and GS supporting evidence: *CHSY1*, *CD80*, *KTELC1*, *IGSF3*, *VAV3*, and *PHLPP1*. They play roles in catalytic activity (*CHSY1* and *PHLPP1*) and signal transduction (*VAV3*). However, the top 10 ADG-related genes according to GS were completely different from these genes identified based on the GS and QTL evidence in both adipose and muscle. In adipose, the top 10 genes positively correlated with ADG were *DHX32*, *FAM160B1*, *ZNF208*, *LETMD1*, *MAPK10*, *COIL*, *Tex35*, *MT-ND4L*, *FOCAD*, and *COL4A5*. Among them, *MAPK10*, *Tex35*, *MT-ND4L*, and *COL4A5* were shown to be downregulated in the Chinese breeds. Two single-nucleotide polymorphisms in *SREBF1* have been found to be associated with ADG in bovine [36]; interestingly, the expression of this gene’s homolog, *SREBF2*, in this study was negatively correlated with ADG (Pearson’s correlation, *r* = −0.62) and positively correlated with FGR (*r* = 0.60).

Using the same method, four genes were found to be correlated with FGR with QTL and GS evidence, namely, *MAPKAPK2*, *LPXN*, *SLC6A17*, and *SEMA4A*, among which *LPXN* was also upregulated in the indigenous breeds. The top 10 genes in terms of the level of GS for FGR in adipose were *LDHA*, *CCL3*, *HEBP1*, *HLA-DRB1*, *CPM*, *TREM2*, *ADAM8*, *CCR1*, *LCP1*, and *DCSTAMP*. All of these were found to be upregulated in the indigenous breeds. In contrast, in muscle, no genes with QTL and gene significance support were found. The top 10 genes in terms of gene significance for FGR were *CILP*, *DEFB1*, *NTRK3*, *SLC12A4*, *HESl-A*, *NELFCD*, *LY6F*, *PI16*, *NNAT*, and *RASA3*.

Here, we discuss together the properties of meat and carcass quality that are representative of YY, namely, being superior in YY than in Chinese breeds, such as the lean meat content (BLMW, FLMW, CLMW, HLLMW, FLMP, BLMP, CLMP, and HLLMP), bone content (FLBW, FLBP, HLBW, CBW, CBP, and BBP), PUFA, and SFA (18:2 and 18:0), among others (HLW, CSAF, and LWA). All of these traits were significantly positively correlated with the coexpression modules AM9, AM15, and AM16, and weakly positively correlated with AM23, AM10, AM13, AM14, AM11, and AM12. AM9 includes an abundance of genes associated with embryonic organ development, which has been reported to differ between lean and obese resistant swine [37]. One gene from AM9, *EYA1*, eyes absent homolog 1, which was downregulated in the Chinese breeds, interacts with several other proteins, including a group known as the SIX proteins, to activate and inactivate genes that are important for normal development [38]. However, its role specifically in adipose tissue still needs investigation.

The protein composition of the extracellular matrix (ECM) changes over time, which is of crucial importance for the function of lipid metabolism of adipocytes in adipose tissue [39]. ECM remodeling is mediated by a balanced complement of constructive and destructive enzymes together with their enhancers and inhibitors. ECM remodeling is an energy-intensive process regulated by insulin, by energy metabolism, and by mechanical forces. Several collagen genes have been reported to be correlated with obesity [40]. This is interesting given that the following genes were included among the hub genes of AM9, *COL4A5* and *COL4A6*, which encode proteins of the collagen alpha(IV) chain; *COL4A5* is also associated with a QTL for intramuscular fat content and *COL4A6* was shown to be downregulated in the Chinese breeds. Another four collagen genes, *COL6A2*, *COL6A4*, *COL6A4*, and *COL8A1*, were also downregulated in the Chinese breeds in adipose tissue. The collagen genes have been reported to be highly expressed in breeds with lower IMF [22], which is consistent with our results. A gene in AM9, *ADAMDEC1*, a disintegrin and metalloprotease-like decysin 1, which belongs to the disintegrin family, also displayed differential expression. ADAM7 and ADAM8, two members of the ADAM family, were also differentially expressed between the indigenous and YY breeds. These results suggest that ECM remodeling plays a key role in the difference in adipose tissue between the two groups of breeds.

One of the hub genes in AM9, *ALDH18A1*, is a member of the aldehyde dehydrogenase family and encodes a bifunctional ATP- and NADPH-dependent mitochondrial enzyme, which catalyzes the reduction of glutamate to delta1-pyrroline-5-carboxylate, a critical step in the *de novo* biosynthesis of proline, ornithine, and arginine. Loss of *ALDH18A1* function is associated with larger cellular lipid droplets [41]. Consistent with our results, the level of expression of members of the aldehyde dehydrogenase family is positively correlated with lean meat and bone traits.

The hub genes in the coexpressed modules other than AM9 include many interesting examples. *ADIPOQ*, *VLDLR*, and *SOD1* are hub genes in AM15. *ADIPOQ* is an important adipokine involved in the control of fat metabolism and insulin sensitivity, with direct antidiabetic, antiatherogenic, and anti-inflammatory activities. *VLDLR*, very low-density lipoprotein receptor, contributes to adipose tissue inflammation and mediates VLDL-induced lipid accumulation and induction of inflammation and ER stress in adipocytes and macrophages [42].

Here, we focus on the properties of meat and carcass quality that are representative of Chinese breeds, namely, higher in Chinese breeds than in YY, such as fat percentage (FLFP, BFP, CFP, and HLFP) and fat thickness (IMF, BFT, LFT, and AFT). Seven genes (*SERINC5*, *CCDC88B*, *NUDT22*, *SCPEP1*, *KIF3C*, *WZSP016177*, and *LBH*) and one gene (*FAM151B*) were found to be related to IMF using evidence from both QTL and GS in adipose and muscle, respectively. Among the coexpressed modules, AM19 and AM6 are significantly correlated with the fat percentage and thickness traits. The KEGG pathway and GO analyses revealed that AM19 is mainly associated with the oxidation–reduction process and catalytic activity, including the pyruvate metabolic process, sulfur compound metabolic process, and purine ribonucleoside metabolic process. These metabolic processes, such as the pyruvate metabolic process, produce Acetyl-CoA, which is the material for fatty acid biosynthesis. This enrichment result is consistent with the positive correlation between AM19 module eigengene and fat percentage and thickness traits. The hub genes participate in fatty acid biosynthesis (*ACACA*), the tricarboxylic acid (TCA) cycle (*ACO1*, *ME1*, *NNT*), transportation (*FABP7*, *SLC12A1*, *SLC2A5*), fatty acid metabolism regulation (*LEP*), transcription (*TCEB3*), and peroxide reduction (*TXNRD1*, *GSS*, *GSR*), among others.

Malic enzyme (encoded by *ME1*) is involved in the TCA cycle to supply NADPH and to transport acetyl-CoA from mitochondria to the cytosol for the biosynthesis of FAs [43]. Its functions in glucose metabolism also contribute to the initial steps of lipogenesis [19]. ME1 mRNA was more abundant in the Chinese breeds than in YY in adipose tissue in our study, which is consistent with the biological function of this gene [16,44–46]. *ACO1* (aconitase 1) functions as an essential enzyme in the TCA cycle and interacts with mRNA to control the level of iron inside cells, related pathways including glucose/energy metabolism, and carbon metabolism [47]. *NNT* (nicotinamide nucleotide transhydrogenase) couples hydride transfer between NAD(H) and NADP(+) to proton translocation across the inner mitochondrial membrane.

Leptin is secreted almost exclusively by fat, and serves as a major ‘adipostat’ by repressing food intake and promoting energy expenditure [48]. Robert *et al*. noted higher leptin mRNA in fat pigs than in lean ones [49], which is in agreement with our study, in which LEP was shown to be upregulated in fat indigenous breeds compared with that in lean YY. The leptin receptor is located in the brain, where nervous regulation of food intake is carried out [50].

Another module, AM6, has similar significance levels regarding its association with fat percentage and thickness compared with AM19, but their functions are markedly different. GO analysis revealed that AM6 is abundant in genes associated with intracellular signal transduction, cytoskeleton organization, and immune system process. KEGG pathway analysis revealed that the most represented pathways are those for phagosome, chemokine signaling pathway, lysosome, cytokine–cytokine receptor interaction, and regulation of actin cytoskeleton. It is known that adipose tissue is an important endocrine organ, which produces and releases adipokines and hormones, including leptin, adiponectin, estrogen, and palmitoleate [6]. These factors, through intracellular signal transduction and chemokine signaling pathway, play critical roles in the maintenance of metabolic homeostasis, which is associated with obesity, and are also potentially involved in swine adipose deposition.

Based on the evidence from QTL and GS in adipose, one gene, *TAF8*, is correlated with PUFA; three genes, *C1ORF70*, *TMEM88B* and *SDF4*, are correlated with lean meat percentage; and one gene, *RGSL1*, is correlated with estimated carcass lean content. Seven (*STX12*, *FAM108C1*, *SEMA6A*, *COMMD10*, *WZSP015663*, *C10ORF46*, *MAD2L1BP*) and two (*ORAOV1*, *XPO5*) genes are correlated with AFT in adipose and muscle, respectively. There are also several genes related to backfat at the tenth rib, back waist fat percentage, and drip loss, among others (S17 Table).

For MBL, the modules significantly correlated with this trait are AM5 and AM6, with the former being more specific to MBL, the latter also being correlated with other traits. GO analysis showed that AM5 genes are mainly associated with immune response (*Staphylococcus aureus* infection, complement and coagulation cascades, phagosome, B-cell receptor signaling pathway, and NF-kappa B signaling pathway, etc.), heterocycle catabolic process (organic cyclic compound catabolic process), and regulation of the actin cytoskeleton. The enrichment analysis results of AM5 and AM6 are similar to those of the DEGs upregulated in adipose in Chinese breeds, indicating that these modules positively correlated with traits harbor more candidate genes that potentially regulate these traits.

In this study, the adipose and muscle tissues were subjected to a comparative analysis of the correlation between their transcriptome and the phenotypic traits. Interestingly, the factors expressed in adipose tissue had a higher correlation to the meat and carcass traits than those in muscle. Most of the lipid metabolism throughout the body takes place in adipose tissue, liver, and skeletal muscle. As an energy source, lipid is stored in adipose tissue. *De novo* fatty acid synthesis also mainly takes place in this tissue. Furthermore, as an endocrine organ, adipose tissue also produces many lipid hormones, including adipocytokines, peptide hormones, and resistin, which influence whole-body metabolism, including that of muscle tissue [51]. Indeed, the upregulated genes in adipose tissue of the Chinese breeds encode proteins involved in many signaling transduction and cell communication-related pathways.

The genes upregulated in muscle tissue in the Chinese breeds are mainly involved in the mitochondrial compartment and oxidoreductase activity, which is concordant with a previous finding that primitive pig breeds have greater oxidative capacity than selected breeds [52].

Among the DEGs that were shared among most of the breeds, four genes were differentially expressed in both adipose and muscle [*MT-ND4L*, *WZSP007035*, *GJA9*, and *MT-ND4L* (WZSP005049)], with the same direction of differential expression (S11 Table). MT-ND4L is a subunit of NADH dehydrogenase, which is the component of the electron transport chain that is responsible for the oxidative phosphorylation process. The *ND4L* gene, which is upregulated in indigenous breeds when located in the mitochondrial genome, but downregulated when from the nuclear genome, may play a key role in the process of fatty acid metabolism. The *GJA9* gene encodes a connexin involved in the formation of gap junctions, intercellular conduits that directly connect the cytoplasm of contacting cells [53], which may influence the transport of FA. To expand the candidate genes that were differentially expressed between the two breed groups across most breeds, if we set the number of common breeds to three, more genes emerge. *PM20D1*, a secreted enzyme that can augment energy expenditure [54], was found to be downregulated in four and five indigenous breeds in adipose and muscle, respectively, which implies that the energy consumption in indigenous breeds is lower than that in YY. *SLC2A5* (solute carrier family 2), facilitated glucose transporter member 5, was found to highly expressed in indigenous breeds across more than three breeds in both tissues, which should provide abundant glucose for use in FA synthesis (S11 Table). In addition, *CCL24*, which encodes a chemokine precursor and was found to be upregulated in indigenous breeds, is related to immune responses. Finally, APOOL (apolipoprotein O-like), which encodes a component of the MICOS complex, a large protein complex of the mitochondrial inner membrane that plays crucial roles in the maintenance of crista junctions, inner membrane architecture, and the formation of contact sites to the outer membrane [55], may be involved in FA metabolism.

In this study, two sets of evidence, on GS and QTL associations, provided us with a large number of genes that potentially contribute to pig meat, carcass, and production-related traits. Genes with higher levels of GS are not necessarily the DEGs, but have high correlations with the trait in question, so this approach provides a greater number of genes related to the specific traits, including those that are not DEGs [56]. These genes should be considered as interesting candidate factors affecting meat and carcass quality, but further studies are needed to resolve the effect of each gene, given that RNA-seq analysis cannot distinguish causal genes from those genes whose expression varies depending on the expression level of other regulating genes.

Although seven Chinese indigenous breeds have been used in this study, only one Western breed was compared. Considering the large difference between these two breeds, it will be interesting to determine whether the modules still significantly correlate with one particular trait after YY has been removed. As expected, the Z_*summary*_ values of the modules from those breeds without YY were all >10, which indicates strong preservation among them, except one module in adipose tissue (S15 Fig). The correlations between the module eigengenes and traits after removing YY were preserved compared with those with YY (S16 and S17 Figs), although the correlations in adipose varied more than those in muscle. Despite this, the adipose modules were still clearly grouped into two classes, which is consistent with the reference modules. However, because only one European breed was used, the variation among the European breeds cannot be ruled out in this study. In future research, more Western breeds, such as Landrace and Duroc, should be included to reduce this variation.

## Materials and methods

### Animal samples and phenotypes

Seven Chinese indigenous breeds, CH, NJ, TP, QY, WJ, YC, and YN, and one introduced Western breed, YY, were raised in Sichuan Animal Science Academy, Jianyang, Sichuan Province, China. These eight pig breeds are all domestic pigs and our study did not use tissues of endangered or protected wildlife. All animals were fed the same diet and three individuals in each group were slaughtered at 6 months of age by a conventional and humane procedure. Specifically, the pigs were killed by electrocution and immediately hoisted for bleeding, dehaired, and then the carcass was dissected. The LD muscle tissues at the 12^th^ rib and the subcutaneous adipose tissues of the back were collected and snap-frozen in liquid nitrogen for the extraction of total RNA. All methods in this study were conducted in accordance with the Regulations for the Administration of Affairs Concerning Experimental Animals (Ministry of Science and Technology, China, revised in June 2004). All experimental protocols were approved by the Institutional Animal Care and Use Committee in Sichuan Animal Science Academy (permit number: XKY-S20140604).

For the same animals on which RNA sequencing was performed, a total of 101 traits related to growth performance, carcass quality, meat quality, amino acid composition, and FA composition in muscle were also measured. In brief, the carcass composition traits were determined according to the methods described by Xiao *et al*. [57]. All meat quality trait measurements were conducted by the methods described by Shen *et al*. [58].

PCA analysis for the phenotypic trait data was performed using the *princomp* function in the R platform. The statistical analysis and production of the graphs used in this study were carried out on the R platform [59].

### RNA isolation and sequencing

Total RNA was extracted using Trizol reagent (Invitrogen, USA), in accordance with the manufacturer’s instructions. RNA quality was assessed by electrophoresis on a 1% agarose gel and with an Agilent 2100 Bioanalyzer (Agilent, USA). mRNA was isolated from total RNA using oligo(dT) magnetic beads and disrupted into short fragments of about 300 bp. Paired-end (PE) libraries were prepared according to the Illumina paired-end library preparation protocol (Illumina, USA). PE libraries were sequenced on an Illumina Hiseq 2000 sequencing system to generate 2 × 125 PE reads at Beijing Genomics Institute at Shenzhen.

### RNA-seq mapping and differentially expressed genes

After the low-quality reads had been trimmed and the adapters had been removed, the clean RNA-seq reads were mapped to the reference genome using Tophat2 (version 2.0.13) with default parameters [60]. The genome sequence and gene structure annotation data of Wuzhishan pig and *Sus scrofa* (strain: Duroc) were downloaded from http://gigadb.org/dataset/100031 and Ensembl, respectively. The unique mapped reads were used to calculate the number of reads that mapped to every gene model using HTseq [61]. Multiple isoforms that belong to one gene were filtered, with the longest isoform being retained as representative of the group. The edgeR package [62] was used to detect the DEGs, which were defined as those with fold change >2 and False Discovery Rate (FDR) <0.1.

### Functional annotation of the WZSP gene

The functions of WZSP’s protein-coding genes were assigned based on the best match derived from alignments to proteins annotated in the NR [63] and SwissProt [64] databases using BLASTP with an E-value <1e^−5^. The KEGG [65] pathway was annotated using the KAAS [66] web service version 2.0, BBH method, by searching against a representative set plus all available mammalian species with the default parameters. We annotated motifs and domains using InterProScan [67] (version 5.13) by searching against the InterPro [68] database (version 52.0). Descriptions of gene products including Gene Ontology [69] were retrieved from InterPro.

### Enrichment analysis

Enrichment analysis for the DEGs was carried out based on an algorithm presented by GOstat [70], with the whole annotated gene set as the background. The *P* value was approximated using the chi-square test. Fisher’s exact test was used when any expected value of count was below 5. The FDR with the Benjamini–Hochberg method was calculated to adjust for multiple testing [71].

### qPCR validation of DEGs

Using the same samples for RNA-seq, qPCR was performed on the aCFX96 Real-Time PCR Detection System (Bio-Rad) using SYBR® Green Real-time PCR Master Mix (Takara, China). The PCR primer sequences are shown in S8 Table. Porcine beta-actin was used as an endogenous control gene. The 2^−ΔΔCt^ method was used to determine the relative mRNA abundance for the surveyed samples.

### DEGs compared with the QTL database

Pig QTL data were downloaded from Animal QTLdb release 29 [72]. A total of 1,672 unique trait locations were selected with the class names “meat and carcass” and “production,” labeled “significant,” and with a *P*<0.05. The coding sequences of Wuzhishan were aligned to the genome reference of *Sus scrofa* to determine the location of the genes from the former. A QTL gene was defined as one with a ratio of the overlapping region to the gene or the QTL region greater than 50%. In total, 3,018 genes in the Wuzhishan genome were defined as QTL genes, among which 1,764 were related to meat, carcass, and production traits. LOLA package [73] is used to calculate the enrichment of the QTL regions in the DEGs using all genes in the WZSP genome as a universal set.

### Weighted gene coexpression network analysis

Coexpression networks were constructed using the WGCNA package [26] separately for adipose and muscle tissues. All of the genes with sum rpkm>5 (14,427 and 13,744 in the two tissues, respectively) were retained for network construction. The raw RPKM values were log2-transformed and then normalized to mean 0 and variance 1 for each column and row. The signed adjacency matrix was calculated using Pearson’s correlation with power β equal to 14 for the two tissues. The topological overlap matrix was calculated based on the adjacency matrix and was then converted into a dissimilarity matrix. Using this dissimilarity matrix, the gene tree was produced by the average linkage hierarchical clustering method. Gene modules were generated using the dynamic tree cut method with height cut-off 0.995, deepSplit 2, and minimum module size 30. Two modules were merged if they had a correlation >0.8 (S6 Fig). Modules were assigned colors and are represented by the color names. Module eigengenes were defined as the first principal component of each module. Within each module, the intramodular connectivity of each gene was measured using two statistics: MM and soft connectivity (K_IM_). MM was defined as the correlation of the expression and module eigengene (ME). K_IM_ was defined as the sum of all pairwise adjacencies of a gene to all other genes in the module. Gene significance was defined as the correlation of the expression and the phenotypic traits.

To validate the robustness of the modules’ correlation to the particular traits, we applied the following two methods. 1) We used Z_*summary*_ statistics to measure the preservation of the modules from the data with YY removed compared with the reference modules constructed from all data. Preservation can be interpreted as the degree to which one module in the test data can be covered by one module in the reference network [74]. 2) The correlations between the module eigengenes and traits were recalculated after removing YY from the expression data.

## Supporting information

S1 Fig Heatmap of the phenotypic traits. Each row represents one trait and each column represents one pig breed. Blue indicates a lower value and red indicates a higher value. Similar traits have been clustered together, as indicated by the group name on the right. The asterisks beside the trait abbreviations indicate the significance of the difference between the Chinese breeds and Yorkshire. * *P*<0.05; ** *P*<0.01; *** *P*<0.001.
(TIF)

S2 Fig The correlation between phenotypic data and the first three principal components. The phenotypic abbreviation follows the colon on the left. Blue represents a negative correlation and red represents a positive one. A more intense color represents a higher correlation value. In the cell, the number outside the parentheses is Pearson’s correlation value and the number within the parentheses is the significance of the correlation. Asterisks before the traits indicate the significance of the difference between the Chinese indigenous breeds and Yorkshire. * *P*<0.05; ** *P*<0.01; *** *P*<0.001.
(TIF)

S3 Fig Sample clustering based on Pearson’s correlation. A: adipose tissue; M: longissimus dorsi muscle tissue.
(TIF)

S4 Fig Pearson’s correlation between the replicates of each sample group. A: adipose tissue; M: longissimus dorsi muscle tissue.
(TIF)

S5 Fig The expression of five genes validated by qPCR. (A) The qPCR relative expression (y-axis) was positively correlated with transcriptome RPKM (x-axis). Pearson’s correlation *r* = 0.5, correlation test *P* = 2.5e^−15^. The R square for the linear regression is 0.25. The qPCR relative expression level was multiplied by 10,000 and log2-transformed. The RPKM had 1 added to it and was then log2-transformed. (B) Most of the DEGs exhibited consistency in their expression ratio between qPCR (y-axis) and transcriptome RPKM (x-axis) when the Chinese breeds were compared with Yorkshire. The red points represent the DEGs between Chinese breeds and Yorkshire detected by transcriptomic analysis. Black represents the genes without differential expression. In total, 72% of the DEGs were consistent in terms of the direction of differential expression, with 17 being consistently highly expressed in Chinese breeds and 9 being highly expressed in Yorkshire.
(TIF)

S6 Fig Gene dendrogram and module colors of the two tissues. (TIF)

S7 Fig Heatmap of the module eigengene values. (TIF)

S8 Fig Module quality of the adipose (left) and muscle (right) coexpression network modules. (TIF)

S9 Fig Module conservation analysis. MedianRank (A and C) and Z_*summary*_ (B and D) were calculated using the modulePreservation() function in the WGCNA package for adipose (A and B) and muscle tissues (C and D). These two measurements give the level of conservation between the adipose and muscle coexpression networks.
(TIF)

S10 Fig Module overlap between adipose and muscle tissues. The upper number in the cell is the number of genes overlapping between the two modules. The lower number in the cell is the P value from Fisher’s exact test. The intensity of color of the cell is the minus log10 of the P value. The number after the colon is the number of genes in the module.
(TIF)

S11 Fig Correlation matrix of adipose coexpression network module eigengene values and phenotypic traits. (TIF)

S12 Fig The significantly correlated coexpression modules and traits in adipose. (TIF)

S13 Fig Correlation matrix of muscle coexpression network module eigengene values and phenotypic traits. (TIF)

S14 Fig Muscle modules MM16 and MM3 are significantly correlated with the phenotypic traits of proline content and fat weight. (A) The expression and eigengene values of modules MM16 and MM3 have opposite correlations with the phenotypic traits. Eig: eigengene, His: histidine, CBW: carcass bone weight, BBP: back waist bone percentage, CFW: carcass fat weight, NR: number of ribs, Pro: proline. (B) Gene significance of proline is positively correlated with module membership in MM16. (C) Gene significance of carcass fat weight is positively correlated with module membership in MM3. (D) The network of the top 30 genes with the highest module membership in module MM16; only the edges with topological overlap above a threshold of 0.2 are displayed. (E) The network in module MM3; the topological overlap threshold is 0.05. The coexpression networks contain the degree of topological overlap (the size of the vertex), the DEGs (the red color of the vertex), and the QTL association (the blue color of the label).
(TIF)

S15 Fig Distribution of Z_*summary*_ after removing YY. (TIF)

S16 Fig Correlation between module eigengenes and phenotypic traits in adipose and muscle tissues after removing YY breed. (TIF)

S17 Fig Comparisons of the module eigengenes and trait correlations between the reference and the data without YY. (TIF)

S1 Table Phenotypic features. (XLSX)

S2 Table Phenotypic variation between Yorkshire and Chinese indigenous breeds. (XLSX)

S3 Table Summary of the RNA-seq and mapping rate. (XLSX)

S4 Table RNA-seq covered genes. (XLSX)

S5 Table Phenotypic comparisons between YY and Chinese indigenous breeds. (XLSX)

S6 Table Phenotypic comparisons between CH and other Chinese indigenous breeds and Yorkshire breed. (XLSX)

S7 Table DEGs related to lipid synthesis, transport, and metabolism. (XLSX)

S8 Table Primer sequences used for qPCR. (XLSX)

S9 Table Enriched GO terms for the Chinese indigenous-biased and YY-biased genes in adipose and muscle tissues. (XLSX)

S10 Table KEGG enrichment of the DEGs from adipose and muscle tissues. (XLSX)

S11 Table Statistics of the DEGs from adipose and muscle tissues. (XLSX)

S12 Table DEGs that overlapped with QTL. (XLSX)

S13 Table GO enrichment for the modules from adipose and muscle tissues, respectively. (XLSX)

S14 Table KEGG enrichment for the modules from adipose and muscle tissues, respectively. (XLSX)

S15 Table The information of hub genes from the significant traits correlated with adipose and muscle modules. (XLSX)

S16 Table GO enrichment of the hub genes. (XLSX)

S17 Table Genes coupling QTL region and phenotypic traits. (XLSX)

S18 Table Top ten genes with the maximum gene significance to the phenotypic traits in adipose and muscle tissues. (XLSX)
