# Supplementary figures and images for: Transcriptomic profiling in muscle and adipose tissue identifies genes related to growth and lipid deposition
Source: PLoS One. 2017 Sep 6;12(9):e0184120. doi: 10.1371/journal.pone.0184120 (PMC5587268; doi:10.1371/journal.pone.0184120)

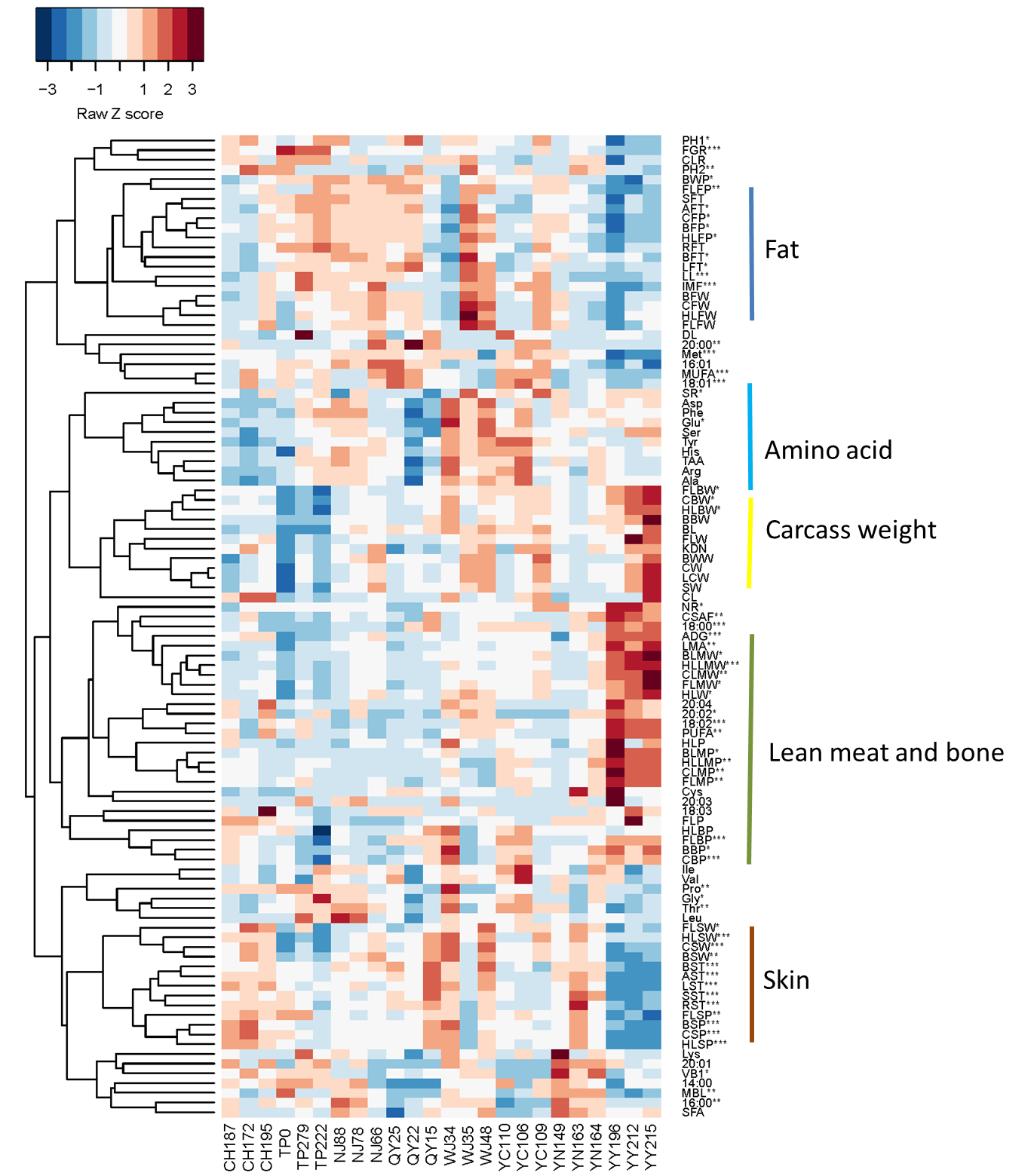

Supplement: S1 Fig — Each row represents one trait and each column represents one pig breed. Blue indicates a lower value and red indicates a higher value. Similar traits have been clustered together, as indicated by the group name on the right. The asterisks beside the trait abbreviations indicate the significance of the difference between the Chinese breeds and Yorkshire. * P<0.05; ** P<0.01; *** P<0.001. (TIF) [file pone.0184120.s001.tif]

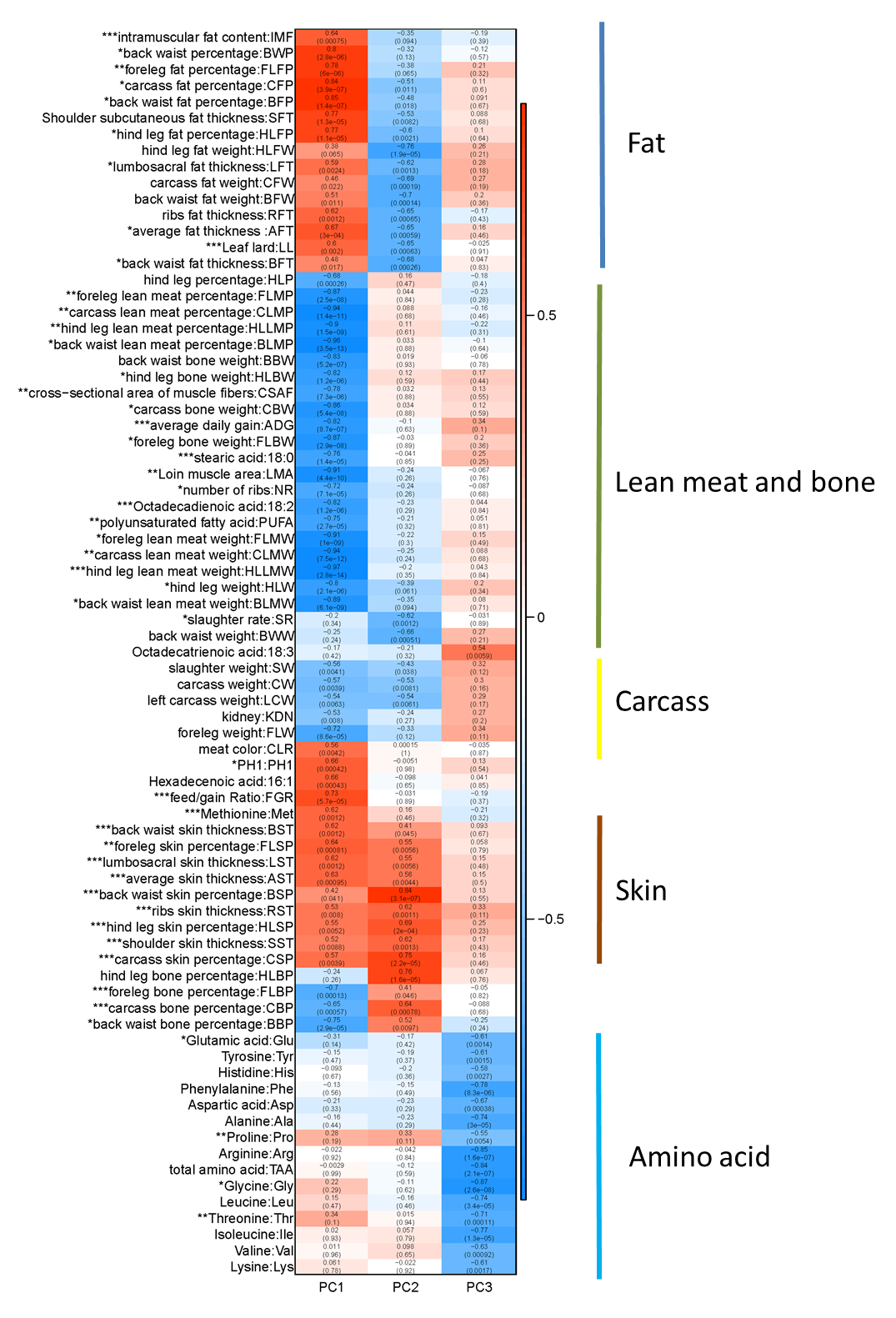

Supplement: S2 Fig — The phenotypic abbreviation follows the colon on the left. Blue represents a negative correlation and red represents a positive one. A more intense color represents a higher correlation value. In the cell, the number outside the parentheses is Pearson’s correlation value and the number within the parentheses is the significance of the correlation. Asterisks before the traits indicate the significance of the difference between the Chinese indigenous breeds and Yorkshire. * P<0.05; ** P<0.01; *** P<0.001. (TIF) [file pone.0184120.s002.tif]

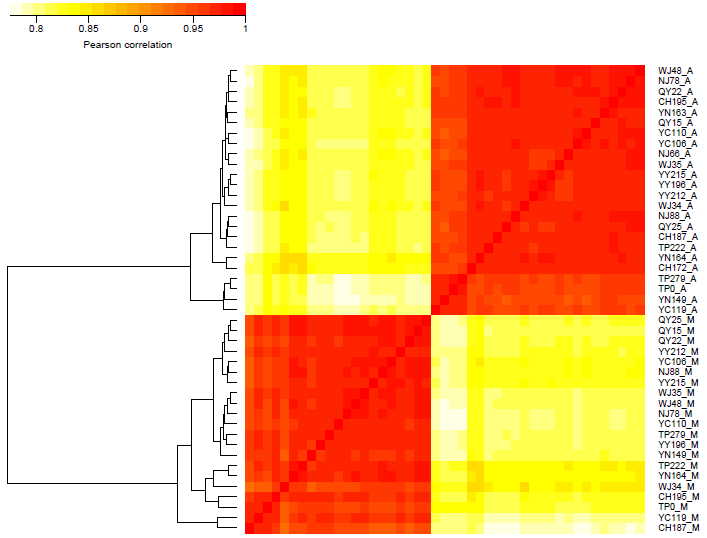

Supplement: S3 Fig — A: adipose tissue; M: longissimus dorsi muscle tissue. (TIF) [file pone.0184120.s003.tif]

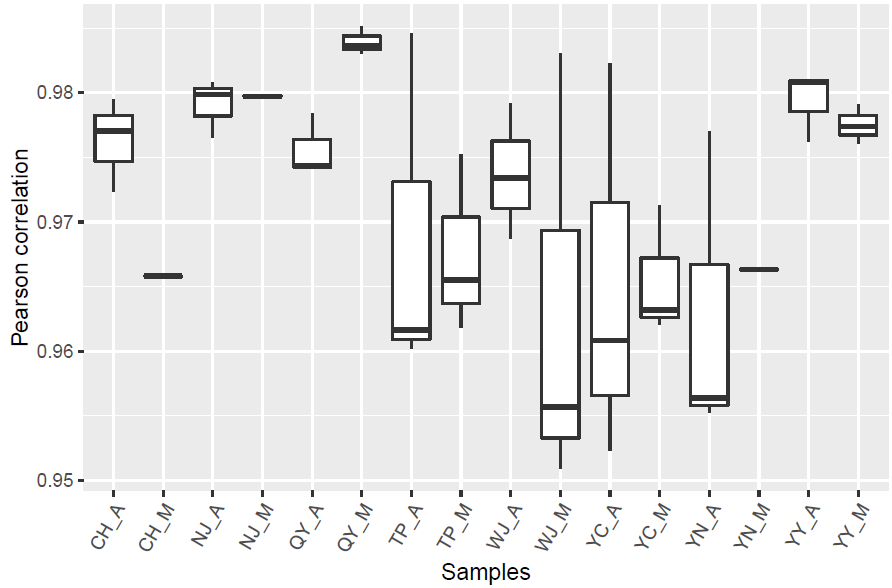

Supplement: S4 Fig — A: adipose tissue; M: longissimus dorsi muscle tissue. (TIF) [file pone.0184120.s004.tif]

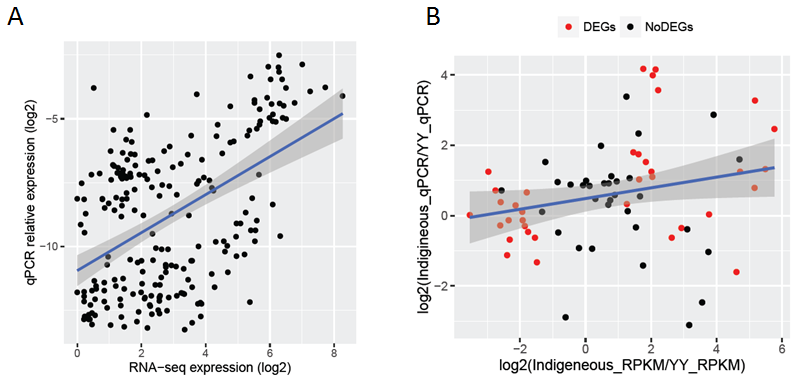

Supplement: S5 Fig — (A) The qPCR relative expression (y-axis) was positively correlated with transcriptome RPKM (x-axis). Pearson’s correlation r = 0.5, correlation test P = 2.5e−15. The R square for the linear regression is 0.25. The qPCR relative expression level was multiplied by 10,000 and log2-transformed. The RPKM had 1 added to it and was then log2-transformed. (B) Most of the DEGs exhibited consistency in their expression ratio between qPCR (y-axis) and transcriptome RPKM (x-axis) when the Chinese breeds were compared with Yorkshire. The red points represent the DEGs between Chinese breeds and Yorkshire detected by transcriptomic analysis. Black represents the genes without differential expression. In total, 72% of the DEGs were consistent in terms of the direction of differential expression, with 17 being consistently highly expressed in Chinese breeds and 9 being highly expressed in Yorkshire. (TIF) [file pone.0184120.s005.tif]

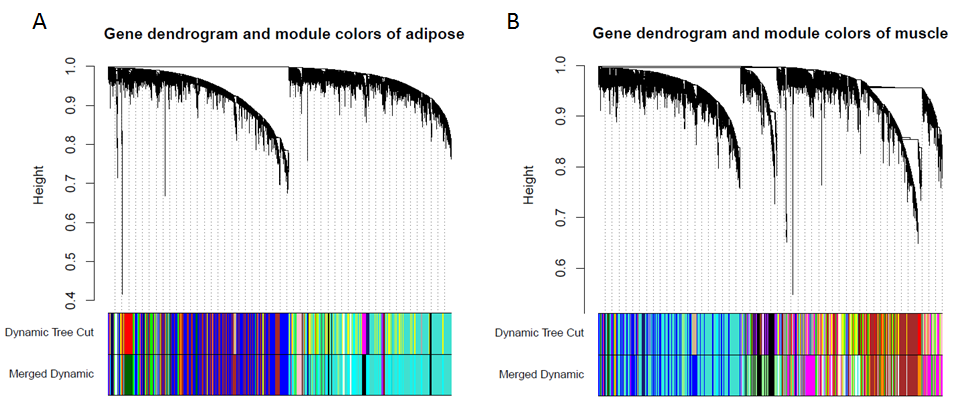

Supplement: S6 Fig — (TIF) [file pone.0184120.s006.tif]

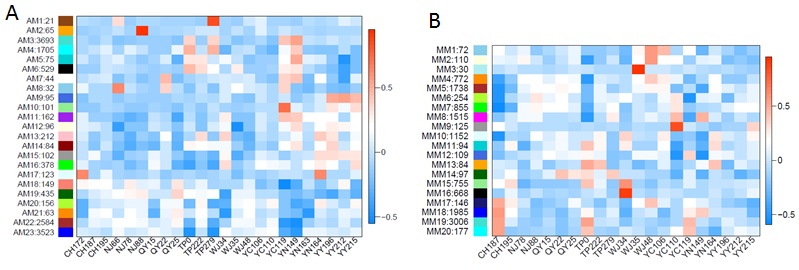

Supplement: S7 Fig — (TIF) [file pone.0184120.s007.tif]

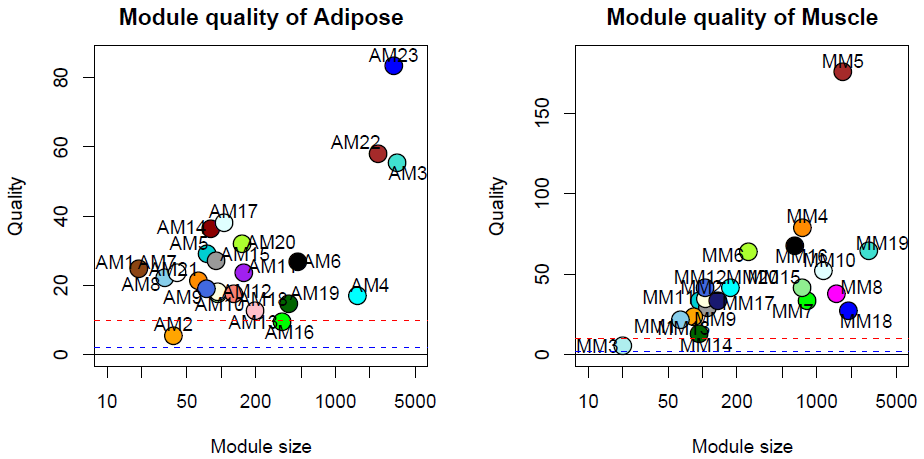

Supplement: S8 Fig — (TIF) [file pone.0184120.s008.tif]

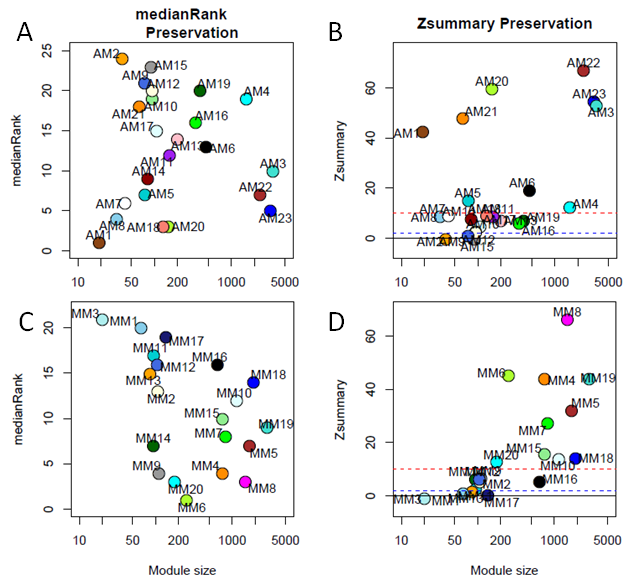

Supplement: S9 Fig — MedianRank (A and C) and Zsummary (B and D) were calculated using the modulePreservation() function in the WGCNA package for adipose (A and B) and muscle tissues (C and D). These two measurements give the level of conservation between the adipose and muscle coexpression networks. (TIF) [file pone.0184120.s009.tif]

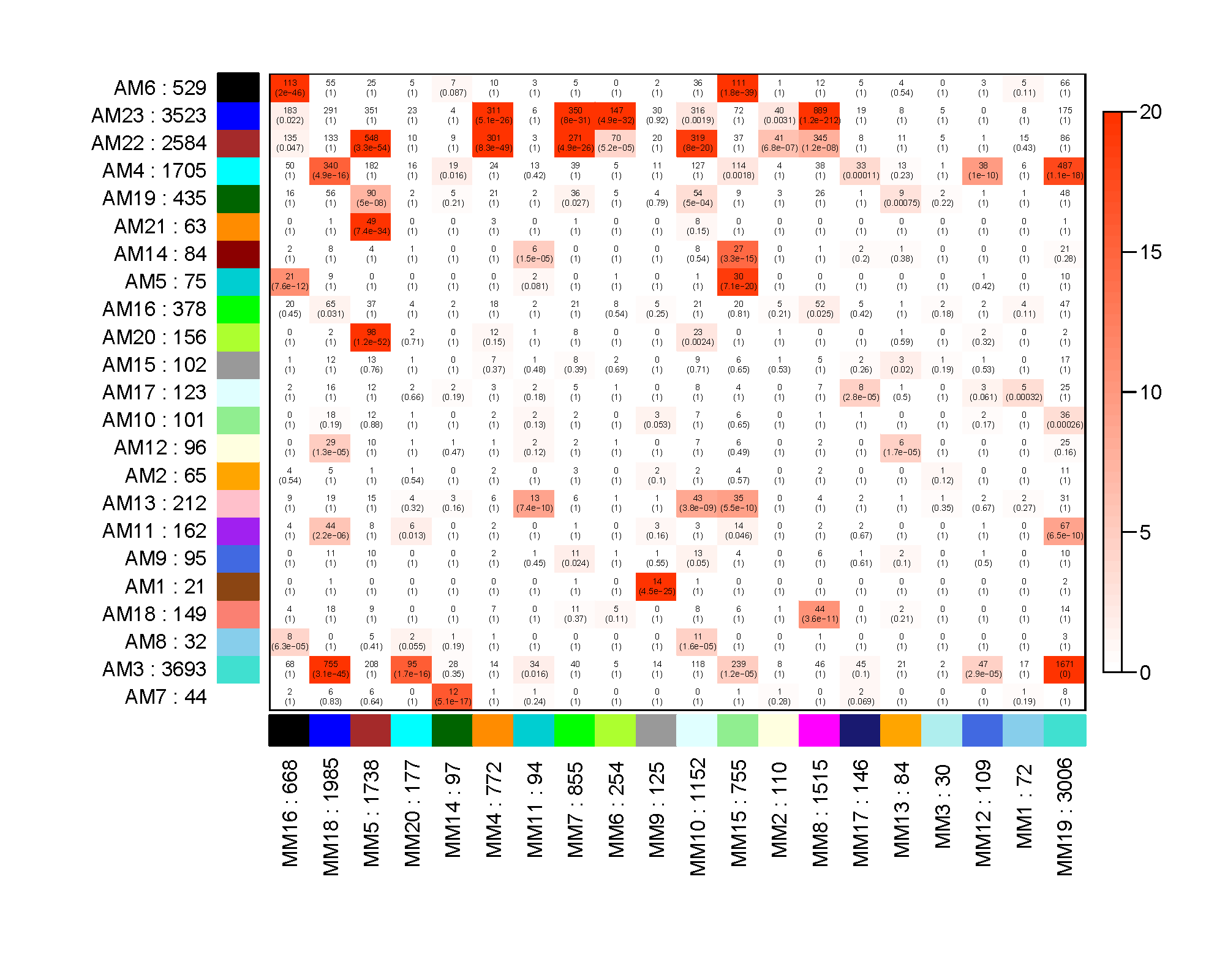

Supplement: S10 Fig — The upper number in the cell is the number of genes overlapping between the two modules. The lower number in the cell is the P value from Fisher’s exact test. The intensity of color of the cell is the minus log10 of the P value. The number after the colon is the number of genes in the module. (TIF) [file pone.0184120.s010.tif]

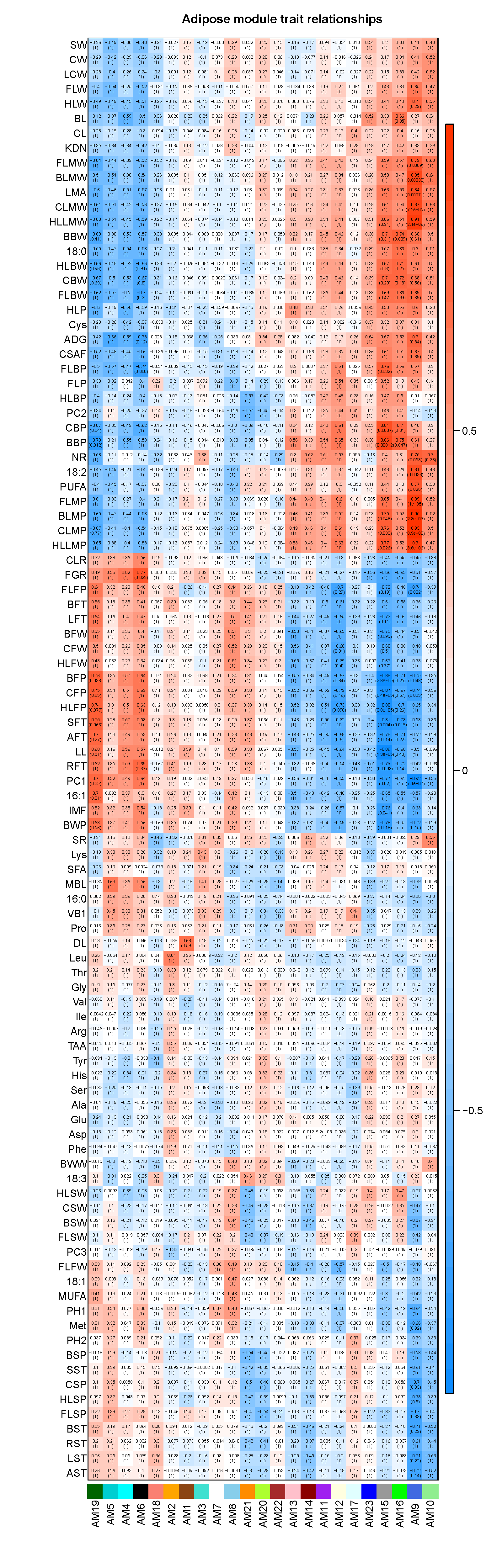

Supplement: S11 Fig — (TIF) [file pone.0184120.s011.tif]

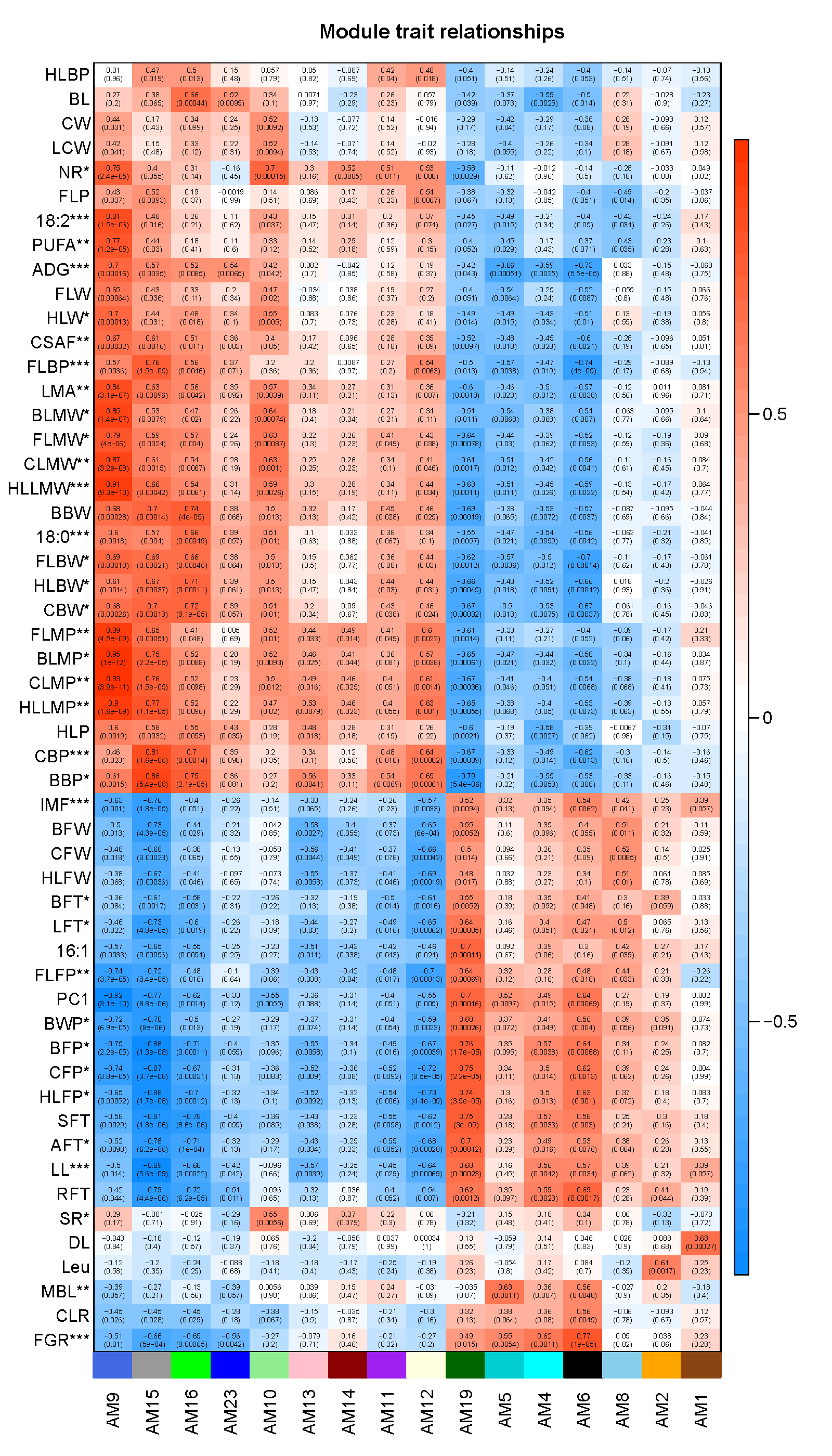

Supplement: S12 Fig — (TIF) [file pone.0184120.s012.tif]

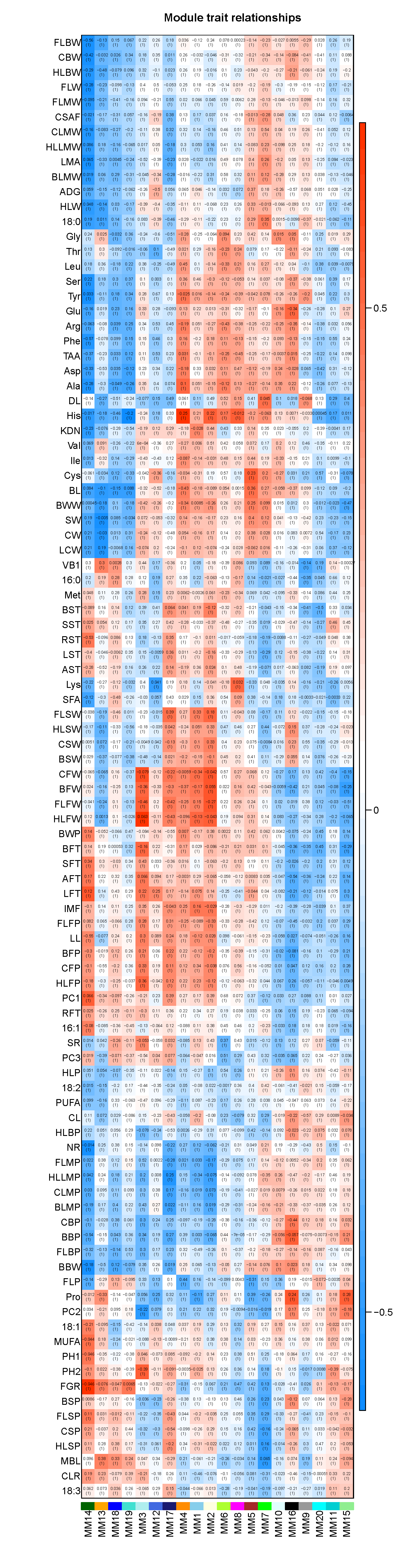

Supplement: S13 Fig — (TIF) [file pone.0184120.s013.tif]

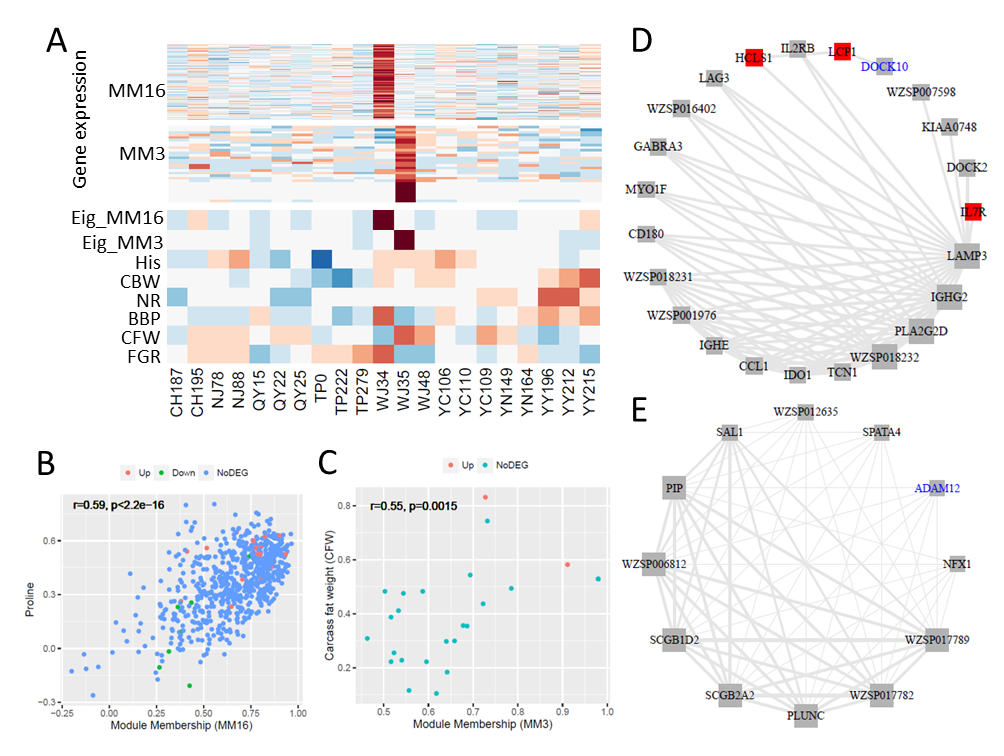

Supplement: S14 Fig — (A) The expression and eigengene values of modules MM16 and MM3 have opposite correlations with the phenotypic traits. Eig: eigengene, His: histidine, CBW: carcass bone weight, BBP: back waist bone percentage, CFW: carcass fat weight, NR: number of ribs, Pro: proline. (B) Gene significance of proline is positively correlated with module membership in MM16. (C) Gene significance of carcass fat weight is positively correlated with module membership in MM3. (D) The network of the top 30 genes with the highest module membership in module MM16; only the edges with topological overlap above a threshold of 0.2 are displayed. (E) The network in module MM3; the topological overlap threshold is 0.05. The coexpression networks contain the degree of topological overlap (the size of the vertex), the DEGs (the red color of the vertex), and the QTL association (the blue color of the label). (TIF) [file pone.0184120.s014.tif]

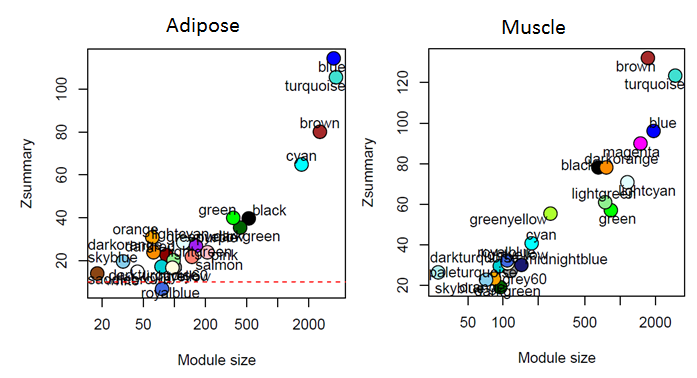

Supplement: S15 Fig — (TIF) [file pone.0184120.s015.tif]

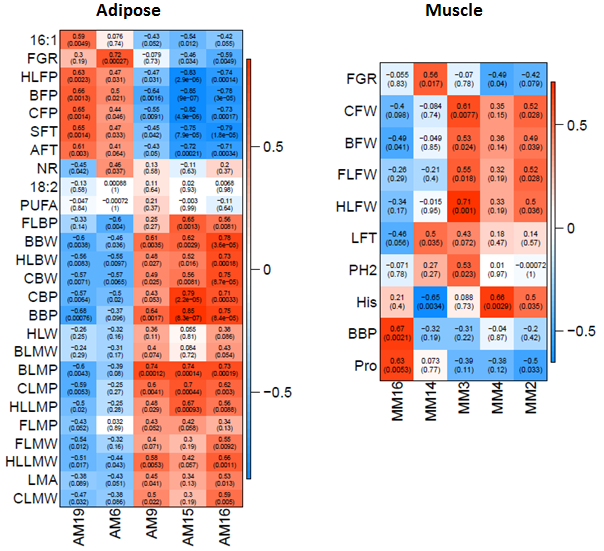

Supplement: S16 Fig — (TIF) [file pone.0184120.s016.tif]

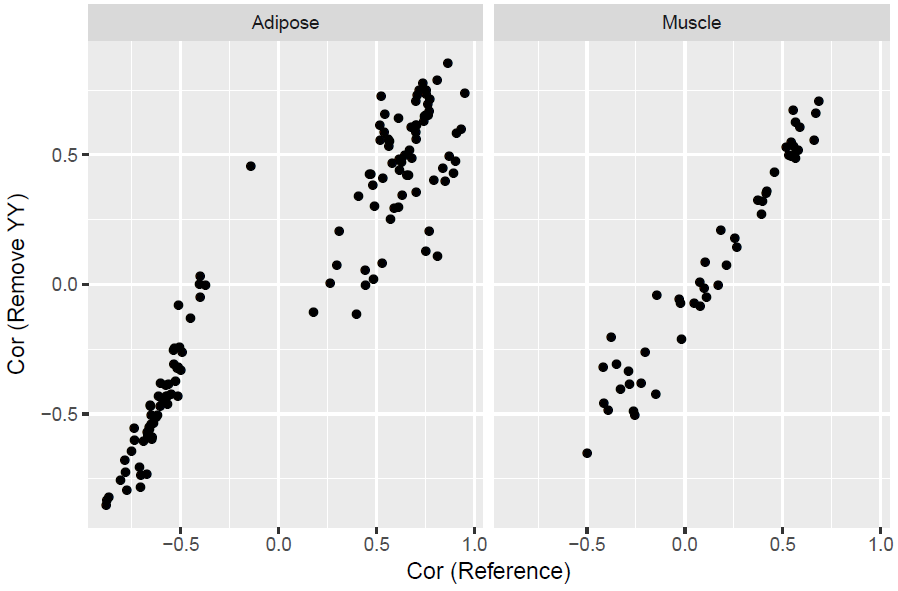

Supplement: S17 Fig — (TIF) [file pone.0184120.s017.tif]
